# Balance-energy of resting state network in obsessive-compulsive disorder

**DOI:** 10.1038/s41598-023-37304-9

**Published:** 2023-06-27

**Authors:** Alireza Talesh, Asghar Zarei, Saeid Yazdi-Ravandi, Ali Ghaleiha, Farshid Shamsaei, Nasrin Matinnia, Jamal Shams, Mohammad Ahmadpanah, Zahra Taslimi, Abbas Moghimbeigi, Reza Khosrowabadi

**Affiliations:** 1 Department of Biomedical Engineering, Tarbiat Modares University, Tehran, Iran; 2 Behavioral Disorders and Substance Abuse Research Center, Hamadan University of Medical Sciences, Hamadan, Iran; 3 Department of Nursing, College of Basic Science, Hamadan Branch, Islamic Azad University, Hamadan, Iran; 4 Behavioral ScienBces Research Center, Shahid Beheshti University of Medical Sciences, Tehran, Iran; 5 Neurophysiology Research Center, Hamadan University of Medical Sciences, Hamadan, Iran; 6 Department of Biostatistics, Modeling of Noncommunicable Disease Research Center, School of Public Health, Hamadan University of Medical Sciences, Hamadan, Iran; 7 Institute for Cognitive and Brain Science, Shahid Beheshti University, Evin Sq., Tehran, 19839-63113 Iran; 8 Biomedical Engineering Faculty, Sahand University of Technology, Tabriz, Iran

**Keywords:** Biomedical engineering, Obsessive compulsive disorder, Computational neuroscience

## Abstract

Stability of the brain functional network is directly linked to organization of synchronous and anti-synchronous activities. Nevertheless, impact of arrangement of positive and negative links called links topology requires to be well understood. In this study, we investigated how topology of the functional links reduce balance-energy of the brain network in obsessive-compulsive disorder (OCD) and push the network to a more stable state as compared to healthy controls. Therefore, functional associations between the regions were measured using the phase synchrony between the EEG activities. Subsequently, balance-energy of the brain functional network was estimated based on the quality of triadic interactions. Occurrence rates of four different types of triadic interactions including weak and strong balanced, and unbalanced interactions were compared. In addition, impact of the links topology was also investigated by looking at the tendency of positive and negative links to making hubs. Our results showed although the number of positive and negative links were not statistically different between OCD and healthy controls, but positive links in OCDs’ brain networks have more tendency to make hub. Moreover, lower number of unbalanced triads and higher number of strongly balanced triad reduced the balance-energy in OCDs’ brain networks that conceptually has less requirement to change. We hope these findings could shed a light on better understanding of brain functional network in OCD.

## Introduction

Obsessive-compulsive disorder (OCD) is a potentially serious mental disorder in which a person experiences uncontrollable and recurring thoughts (obsessions) and behaviors (compulsions) and feels the need to repeat these thoughts and behaviors several times^1^. This illness can be diagnosed by observing a patient's compulsive behavior. It should be noted that OCD is not only described by the symptoms of abnormal thoughts and behaviors. It can also cause cognitive dysfunction of the brain, resulting in complications such as decreased decision-making power, information processing speed, reduced planning power, and impaired visual-spatial skills^2–8^. The severity of the symptoms of obsessive-compulsive disorder may change over time. But if left untreated, this disorder can last for years or decades. People with OCD frequently experience other types of mental illnesses. Statistics show that about three-quarters of adults with OCD are diagnosed with an anxiety disorder at some point in their lives, such as generalized anxiety disorder or panic disorder. Some of the other important comorbidities are impulse-control disorders (55.9%), and substance use disorders (38.6%)^9^.

In addition, the results of previous studies have shown that cognitive dysfunction and abnormal behavior in people with OCD are associated with functional and structural changes in their brains^10–14^. For example, in subjects with OCD, the volume of the Globus pallidus^15^, the caudate nucleus^16,17^, and the overall size of the cortex^18,19^ are reduced compared to healthy controls (CON). Other recent studies have reported an association between dysfunction in the cortico-striato-thalamo-cortical circuits and clinical signs and cognitive functions such as executive functions^20^ and inhibitory control^21^ in people with OCD.

Although changes in brain function in people with OCD have been studied using a variety of neuroimaging techniques, relatively less is discovered using the EEG technique^22^. The importance of neural oscillations has been demonstrated in various perceptual and cognitive tasks. For instance, previous studies have shown that theta band [4–8 Hz] activities increase, and beta band activities [13–30 Hz] decrease in the frontal and frontotemporal regions of the brain^23–25^. Moreover, changes in frontal asymmetry at the lower alpha band, and increase in the complexity of information processing in beta and lower gamma bands at the frontal regions have been reported^26^. Ischebeck and colleagues^27^ found that the frontal power of OCD patients in comparison to CON subjects was more dominant in the left hemisphere at the lower alpha band. During a stimulus–response compatibility task study, overactivity during response preparation was seen in motor regions^28^. Wong and colleagues^29^ investigated the relation of Padua-R, a measure of the severity of OCD-related behaviors, with alpha activity in the frontal and parietal regions. They found that a higher score of Padua-R was accompanied by lower overall activity in the frontal regions.

On the other hand, the globalism view over the past two decades has shown that the brain works as a whole, and the extent of brain damage is more important than its location. In this regard, graph theory is a valuable tool that provides useful information about complex brain networks^30–32^. Graph theory-based techniques can reveal the fundamental characteristics of the brain network's organization and function along with detecting changes in brain disorders^33,34^. Nevertheless, the impact of the links on each other’s has not been well understood. Suppose the x, y, and z regions are connected in a signed brain network. What is the inevitable impact of xy and zy interactions on the sign and weight of the interaction between x and z? It seems that it is not realistic to study the xy interaction independent from the triadic interconnection (xyz) that it lives in.

Recently, triadic interactions were investigated using long-term structural balance theory (SBT). The findings showed that triadic interactions played a very important role in the stability of the signed networks of real-world organizations^35–37^. The mathematical relationships of the SBT method were first proposed by Cartwright and Harry^38^. For the past two decades, this method has been a popular approach in examining the structures of biological, political, and social networks. Analyzing and clarifying the role of balanced triadic interactions in the formation of the global structure of a network is a distinguishing feature of the SBT method. The SBT theory helped to a better understanding of how the tendency to reduce overall stress affects the organization of the signed networks. In particular, the SBT argues that the link between two nodes is strongly influenced by the presence of the third node in a triadic structure. Different types of triads can be categorized into four groups, including strongly balanced T_3_: (+ + +), weakly balanced T_1_: (+ − −), strongly unbalanced T_2_: (+ + −), and weakly unbalanced T_0_: (− − −). The “ + ” and “−“ are the sign of functional connectivity values between the regions (herein: EEG electrodes).

For the brain networks, Moradimanesh and colleagues analyzed the brain triadic interactions to compare the autism spectrum disorder (ASD) group with CONs^39^. They found that balanced and unbalanced triads were over-presented and under-presented in both ASD and CON groups, respectively. Also, they observed that the balance-energy (the summation of the negative of the triads’ weights products is named balance-energy because of its relation to energy and balance of the network (more explanations are given in the structural balance subsection)) of the salience network (SN) and the default mode network (DMN) were lower in ASD, probably indicating the difficulty of flexible behavior. In another study, Saberi and colleagues used the SBT concept for the analysis of brain networks^40^. They introduced a quantitative metric for the global hubness of the network to investigate the impact of negative and positive links and their topology on the network stability. The authors found that the brain network tends to make hubs mainly by negative links and the resulting topology moves the network into a more stable condition. In the same line of research, we thought that brain functional networks of OCDs must also be influenced either by the amounts of negative/positive links or their arrangement in the network (topology). Considering the exaggeration of information processing in OCD, we hypothesized that positive feedback by the tendency of positive links to gather together must be a reason. Moreover, this tendency should be a way to guide the network to a more balanced state.

In this study, three works were carried out for the first time in OCD studies: (1) Triadic interaction analysis using the SBT to compare the stability and triadic structures of OCD signed weighted networks in comparison to the CON ones; (2) Global hubness analysis of OCD and CON networks to find the topological differences between OCD and CON signed weighted networks; (3) The relations of the topological differences to the stability of signed weighted networks of OCD and CON groups. The study steps will be described in the following sections.

## Results

**Figure 1:**
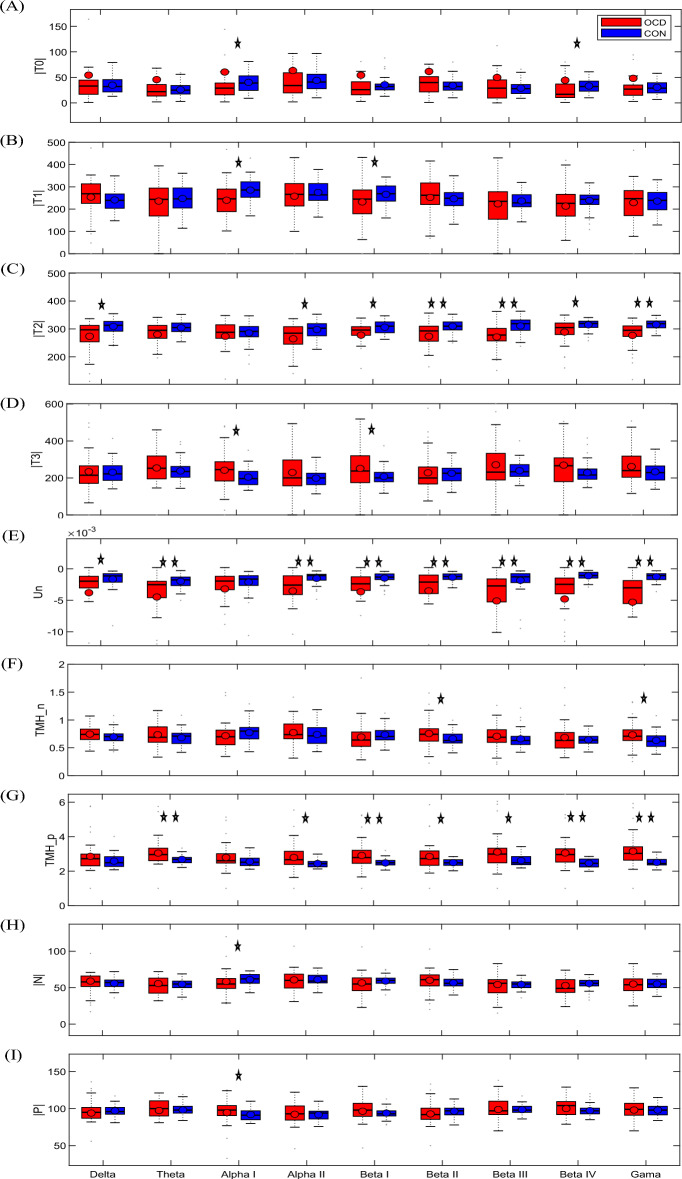
The boxplots of studied measures in nine well-known frequency bands of EEG. The studied measures are the number of triads |T_i_|, the total energy of triads Un, the tendency to make hubs with negative/positive links TMH_n/TMH_p, and the number of negative/positive links |N|/|P|. The circles represent the mean values and the lines within the boxes indicate the median values. The significant differences with *p* < 0.05 and *p* < 0.005 are indicated by one and two stars, respectively. Abbreviations: *OCD* obsessive-compulsive disorder, *CON* control.

Comparison of the total number of positive and negative links between OCD and CON groups demonstrated no significant results in most of the frequency bands as presented in the first and second rows of Table 1 and panels H and I of Fig. 1. All these comparisons were separately carried out in each of the nine frequency bands, i.e., Delta, Theta, Alpha I, Alpha II, Beta I, Beta II, Beta III, Beta IV, and Gama. The significant differences were only observed at the Alpha I band as a smaller number of negative links in OCD patients (*p* = *0.011*, *z* = − *2.53*) and a greater number of positive links in OCD individuals (*p* = *0.011*, *z* = *2.53*). The same pattern was observed for Beta I and Beta IV, however, differences between the groups were not significant. It should be mentioned that after correction for multiple comparison effects using the Bonferroni method, none of the results were significant. Nevertheless, the average value of |N| for all the subjects was approximately 63, and the average value of |*P*| for both healthy and OCD individuals was approximately 90, almost 1.5 times the average number of negative links.

**Table 1:** The Wilcoxon rank-sum test results.

	Delta	Theta	Alpha I	Alpha II	Beta I	Beta II	Beta III	Beta IV	Gamma
|N|	*0.36*	*0.57*	***0.011***	*0.477*	*0.06*	*0.25*	*0.97*	*0.08*	*0.506*
	0.9	− 0.56	− 2.53	− 0.7	− 1.89	1.13	0.03	− 1.74	− 0.66
|*P*|	*0.36*	*0.57*	***0.011***	*0.477*	*0.06*	*0.25*	*0.97*	*0.08*	*0.506*
	− 0.9	0.56	2.53	0.7	1.89	− 1.13	− 0.03	1.74	0.66
|T_0_|	*0.67*	*0.84*	***0.02***	*0.34*	*0.11*	*0.21*	*0.87*	***0.006***	*0.28*
	− 0.41	− 0.2	− 2.2	− 0.94	− 1.57	1.25	− 0.16	− 2.73	− 1.06
|T_1_|	*0.08*	*0.48*	***0.008***	*0.58*	***0.04***	*0.36*	*0.94*	*0.08*	*0.83*
	1.72	− 0.7	− 2.6	− 0.55	− 2	0.9	− 0.08	− 1.76	− 0.21
|T_2_|	***0.006***	*0.07*	*0.92*	***0.04***	***0.04***	***0.001***	***6e-4***	***0.03***	***6e-5***
	− 2.75	− 1.8	− 0.1	− 2.02	− 2.07	− 3.16	− 3.43	− 2.14	− 4
|T_3_|	*0.57*	*0.36*	***0.02***	*0.335*	***0.03***	*0.35*	*0.76*	*0.099*	*0.2*
	− 0.5	0.9	2.26	0.96	2.14	− 0.9	0.29	1.65	1.27
Un	***0.007***	***4e*-*4***	*0.25*	***0.004***	***5e*-*4***	***0.002***	***0.003***	***4e*-*6***	***7e*-*8***
	− 2.7	− 3.5	− 1.14	− 2.89	− 3.45	− 3.07	− 2.94	− 4.58	− 5.38
TMH_n	*0.09*	*0.45*	*0.072*	*0.407*	*0.136*	***0.02***	*0.103*	*0.88*	***0.023***
	1.7	0.7	− 1.79	0.83	− 1.49	2.24	1.63	− 0.15	2.28
TMH_p	*0.15*	***0.003***	*0.124*	***0.008***	***8e*-*4***	***0.026***	***0.011***	***4e*-*5***	***5e*-*6***
	1.4	2.98	1.54	2.65	3.35	2.21	2.52	4.11	4.54

The number of triads |.|, energy Un, the tendency to make hubs with negative/positive links TMH_n/TMH_p, and the number of negative/positive links |N|/|*P*| are statistically compared between OCDs and CONs in nine frequency bands. The *p* and *z* values are in the first and second rows of each cell, respectively. The boldface values are *p-values* smaller than 0.05. The negative/positive sign of *z* shows that that measure is smaller/larger for OCDs in comparison to CONs.

Significant values are in italics.

Then, considering no significant differences in terms of the total number of positive and negative links between the groups, the effect of the topology of the links was investigated by comparison between the global tendency in the network to make positive and negative hubs introduced as the TMH by Saberi and colleagues^40^. The TMH_n and the TMH_p metrics of OCDs were compared with CONs and results are presented in panels F and G of Fig. 1, and the last two rows of Table 1. As demonstrated, despite no significant differences in terms of the number of negative links, the functional links in the OCD group had more tendency to make hubs of negative links at Beta II (*p* = *0.02*, *z* = *2.24*) and Gamma (*p* = *0.023*, *z* = *2.28*) bands. As presented in Table 1, the tendency for making hub with positive links was also significantly more for OCD patients compared to healthy subjects in most of the studied EEG bands at Theta (*p* = *0.003*, *z* = *2.98*), Alpha II (*p* = *0.008*, *z* = *2.65*), Beta I (*p* = *8e-4*, *z* = *3.35*), Beta II (*p* = *0.026*, *z* = *2.21*), Beta III (*p* = *0.011*, *z* = *2.52*), Beta IV (*p* = *4e-5*, *z* = *4.11*) and lower Gamma (*p* = *5e-6*, *z* = *4.54*). The results of the Theta, Alpha II, Beta I, Beta IV, and Gamma band remained significant after correction for multiple comparison effects. As presented in panels F and G of Fig. 1, it seems that positive links in OCDs have more tendency to make hubs. However, it should be noted that the average number of positive links was about 1.5 times the average number of negative links which could potentially lead to an increase in the value of TMH_p compared to the TMH_n. The average values of TMH_p were in the range of 2–3 which are almost 3 times larger than their corresponding TMH_n values.

Subsequently, the number of balanced and unbalanced triads were compared and the structural balance-energy of the networks in OCD and CON groups were examined. The investigation was performed by analyzing the triadic interactions of four types of triads, i.e., strongly balanced T_3_: (+ + +), weakly balanced T_1_: (+ − −), strongly unbalanced T_2_: (+ + −), and weakly unbalanced T_0_: (− − −). The number of triads |T_i_| and the balance-energy Un were two metrics for quantitative comparison between the structural balance of OCDs and CONs functional brain networks. For unbalanced triads, the number of weak (T_0_) and strong (T_2_) unbalanced triads are presented in Fig. 1 (panels A and C), respectively. According to Fig. 1A, the average number of T_0_ varied approximately between 30 and 50. As presented in Fig. 1C, the average number of the strongly unbalanced triad, |T_2_|, approximately varied between 250 and 300 which is almost six times larger than the number of the weakly unbalanced triad. In all nine EEG bands, the average number of T_2_ for CONs was more than the OCDs. The results of the number of the weakly balanced triad, |T_1_|, and strongly balanced triad, |T_3_|, are shown in panels (B) and (D) of Fig. 1, respectively. The average values of |T_1_| were in the range of 200–300. Also, the average number of the weakly balanced triad for the CON group was more than the OCDs in most of the studied EEG bands (except Delta and Beta II bands). The average values of |T_3_| also approximately varied between 200 and 300. It is noteworthy that the brain networks of the OCD and CON groups had opposite behavior in terms of two measures of |T_1_| and |T_3_| in most of the nine frequency bands. In addition, as presented in Fig. 1E, the total energy of triads was negative for both OCD and CON groups in all the EEG bands. While the average values of Un were approximately in the range of − 0.006 to zero. As depicted in panel E, the average values of the Un for OCD group were smaller than the CON subjects in all the nine EEG bands. The results of the statistical comparison between the average number of balanced/unbalanced triads, and their balance-energy are presented in Table 1.

According to Table 1, the numbers of the weakly unbalanced triad for the OCD group were significantly less than the CONs in Alpha I (*p* = *0.02*, *z* = − *2.2*) and Beta IV (*p* = *0.006*, *z* = − *2.73*) bands. Also, the results showed that the numbers of the weakly balanced triad for CONs were significantly more than the OCD group in Alpha I (*p* = *0.008*, *z* = − *2.6*) and Beta I (*p* = *0.04*, *z* = − *2*) bands. The |T_2_| for OCD patients was significantly less than the CON subjects in most of the EEG bands (all *p-values* < *0.04* and all *z-values* < − *2.02*). The statistical results indicated that the number of strongly balanced triads for OCDs was significantly more than CON subjects in Alpha I (*p* = *0.02*, *z* = *2.26*) and Beta I (*p* = *0.03*, *z* = *2.14*) bands. Examining the statistical *z* values showed that |T_3_| had opposite behavior in comparison to other triads, including |T_0_|, |T_1_|, and |T_2_|.

In addition, only the triad T_2_ was significantly different between OCD and CON groups almost in all frequency bands (except Theta and Alpha I). Hence, one may be interested in knowing which links of T_2_ had the highest/lowest occurrence rates for OCDs and simultaneously had the lowest/highest occurrence rate for CONs in the mentioned frequency bands (by considering the definition of “occurrence rate” given in the “Structural balance” section, it can be said that links of T_2_ with the highest/lowest occurrence rate are the most/least frequent links in T_2_. In the next lines, it is explained how to find these links). To answer this question, all the T_2_s of both OCD and CON groups in all nine frequency bands were obtained. From these T_2_s, 816 different T_2_s were found (it should be noted that the total number of T_2_s is much more than 816 because each of these 816 T_2_s is seen for many subjects out of 78 subjects or even for a given subject, one T2 is probably seen in more than one frequency band. Thus, the total number of different T_2_s was 816). These T_2_s offered 2448 links (each triad has three links). For each frequency band, these links were investigated separately. These links were sorted based on their times of presence (or occurrence rate) in T_2_s of all subjects of a given group. The sorting process of links (sorting from the highest rate to the lowest times of presence) was carried out for each group and each frequency band, separately. For each band and group, the links in the top 10% / bottom 10% of the sorting list were considered the links with the highest/lowest occurrence rates. The functional links between F8 and P3 in the Delta band, and T5-C3 in the Beta III band had the highest occurrence rates for CONs and the lowest occurrence rates for OCDs. The F8-P3 was seen 231 times in triads T_2_ of CONs so that in 74.89% of occurrences had positive values and in 25.11% of occurrences had negative values; while this link occurred 210 times in triads T_2_ of OCDs with positive values in 74.28% and negative values in 25.72% of occurrences. The functional link between T5 and C3 was also observed 232 times in triads T_2_ of CONs (78.88% positive, and 21.12% negative); while this link was observed 215 times in triads T_2_ of OCDs (38.14% positive, 61.86% negative). In contrast, the T3-F3 in Delta band and Fz-Fp2 in Beta II were the only links that had the highest occurrence rates for OCDs and simultaneously the lowest occurrence rates for CONs. The T3-F3 link was observed 257 times in triads T_2_ of OCDs (55.25% with positive values, 44.75% with negative values); while this link was seen 189 times in triads T_2_ of CONs (50.8% positive, and 49.2% negative). The Fz-Fp2 was also observed 261 times in triads T_2_ of OCDs (77.77% positive, and 22.23% negative), while this link was observed 174 times in triads T_2_ of CONs (76.44% positive, and 23.56% negative). Having said that, it must be mentioned that only the T5-C3 link showed a significant difference between OCD and CON groups in terms of percentile of appearing as the positive and negative links.

**Figure 2:**
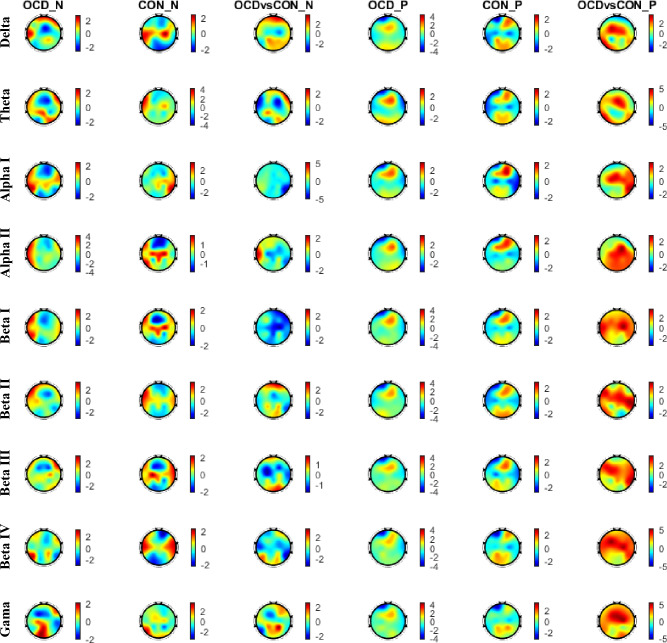
The degree distribution of positive and negative links in nine EEG frequency bands. The “_N”/”_*P*” in the figure indicates that the results are for negative/positive degrees. In the first, second, fourth, and fifth columns, the red and blue colors show the regions of the head with the most and the least degrees, respectively. In the third and sixth columns, the red and blue colors indicate regions with higher and lower degrees for OCDs compared to CONs, respectively.

Moreover, the TMH is a global metric and one may be interested to localize the regions based on their degree distributions. It can help to understand which regions play the main role in differentiating the topology of brain functional networks in OCD versus CON. The positive degree of a given electrode was computed by summing the weights of positive links connected to the electrode and likewise for the negative links. In each frequency band and for each group, the positive and negative degrees were computed for each electrode and averaged over the subjects. Then, the z-scored value of each electrode’s degree was calculated based on the mean and standard deviation of degree values of all 18 electrodes. Subsequently, the z-score values of positive and negative links of OCD and CON groups were compared using the Wilcoxon rank-sum test. As presented in Fig. 2, degrees of electrodes at the Frontal, Central, and Temporal regions showed significant differences between OCD and CON groups despite the similar pattern of degrees in both groups.

In addition, the balance-energy Un for CON individuals was significantly higher than the OCD patients in all EEG bands (except Alpha I) (all *p-values* < *0.002* and all *z-values* < − *2.7*). It should be noted that U_n_ showed similar behavior to the triadic metrics except |T_3_| when comparing OCD with CON groups.

## Discussion

Previous studies have shown that functional associations between the brain regions are significantly changed in OCD individuals^3,41–43^, which could be a potential biomarker for diagnosis of the OCD. However, the characteristics of the changes still require to be well understood. Undoubtedly, amounts of the negative and the positive links as well as their arrangement in the network (topology) could influence the collective behavior of the network and its stability^40,44^. In addition, looking at the exaggerated form of information processing in the Frontal region in OCD^26^, one may think of the effect of positive feedback provided by the regional synchrony to other parts of the brain. Nevertheless, the positive feedback must be in a form that forces the network to a stable state. Therefore, we hypothesized positive links must have a more tendency to gather together to provide positive feedback, while the arrangement of the negative links leads the network to a more unbalanced state. To investigate the hypothesis, the structural balance theory and metrics of TMH were employed^39,40^. The SBT allowed us to study four types of triadic interactions and balance-energy in the OCD network in comparison to the CON. Moreover, the TMH metrics allowed us to perform a comparison between the topology of positive and negative links in the brain functional networks of OCD and CON groups^40^.

To the best of our knowledge, the current study presents the first application of balance theory using EEG so far. In this study, Heider’s balance theory was investigated for OCDs and CONs in different frequency bands of EEG^45^. We aimed to show how the gathering of positive and negative links could influence the balance-energy of the brain functional network in OCDs by over-/under-presenting balanced and unbalanced triads in the network. Since the triadic associations are based on both positive and negative links as well as their topology, therefore, amount of positive and negative links and their topology were compared between the OCD and CON groups as explained in the method section. The amount of positive and negative links was not significantly differentiable between OCD and CON groups, except in the Alpha I band. In the Alpha I band, the |*P*| was significantly greater for OCDs compared to CONs. This means that phase synchrony (resulting in positive links) of brain regions of OCDs is abnormal in this band. We think of such abnormal alpha synchrony could be a reason for the low energy level of the OCD brain, which in turn leads to a more stable state for the OCD brain. The discussions provided in the next paragraphs (about TMH_p and triadic interactions T_2_ and T_3_) offer more evidence for this thinking. Based on the characteristics of OCD disease, we think this stable state is the state providing obsession and compulsive characteristics for OCDs. Positive links individually make their effects on the OCD brain state in the alpha I band. However, if we view positive links through their interactions with positive and negative links (through triadic interactions T_2_ and T_3_) or their arrangement by other positive links (through TMH_p), we could say that positive links play the key roles in many frequency bands for OCD patients in terms of OCD characteristics and keeping the brain in a stable state (we think this state is not appropriate and is related to OCD characteristics). In many frequency bands, the OCDs in comparison to CONs had lower T_1_ and T_2_ and higher T_3_ and TMH_p. These results show that positive links in OCDs have less tendency to associate with negative links and, oppositely, they tend to integrate with positive links. As we said in this paragraph and we will say in the following paragraphs, the lower T_2_ and higher TMH_p and T_3_ lead to lower energy levels and consequently prevent state changes in the OCD brain (again, it is said that the mentioned lower and higher behaviors and consequently lower energy level in OCDs’ brain have resulted from abnormal tendency of positive links for integrating with positive links and repelling the negative ones). As a result, the neuroplasticity of the OCD brain is decreased. From this point of view, it can be said that phase synchronization among different brain regions of OCDs may be leading to repetitive thoughts and compulsive behaviors.

In contrast to |*P*| and |N|, the arrangement of the links showed significant differences, particularly for the positive links. Based on the TMH results, the topology of the negative links was significantly altered in the OCDs at the networks of Beta II and Gama bands; while the arrangement of the positive links was significantly changed (TMH increased) in the OCDs’ networks at all the frequency bands except for Delta and Alpha I. The changes in topology had a larger effect on positive links than those of negative links. Such a tendency to make positive hubs in the OCDs’ brain functional networks could influence the network's stability by changes in triadic associations (offering lower T_2_ and higher T_3_ for OCDs).

Both TMH_n and TMH_p showed negative correlations with Un for both groups. However, the impact of TMH_p (*p-values* < *e-8, r-values* < − *0.73*) was much more than TMH_n (*p-values* < *0.01, r-values* < − *0.37*) for both groups. The TMH_p impact on Un of OCD (*p-values* < *e-14, r-values* < − *0.89*) was much more than that of CON (*p-values* < *e-8, r-values* < − *0.73*) in all frequency bands. Also, the results of Table 1 showed much more TMH_p for OCDs than CONs in all frequency bands. Therefore, it can be said that the positive links play the dominant role in the topology alternation of OCDs, which in turn leads to more stability and less dynamism (less energy Un) of the OCD brain network.

The unbalanced triads T_0_ and T_2_ can change balance-energy and consequently the state of the network by injecting energy into the network. Therefore, although the T_0_ and the T_2_ are less-presented in the network as compared to the T_1_ and the T_3_, they play a key role in the dynamism of the brain functional network^39^. The results of this study also showed that the |T_0_| and particularly |T_2_| were lower for the OCDs compared to the CONs in all the frequency bands (see Table 1). This may introduce fewer requests for a change in the OCD’s network and bring the network to a more stable state as presented in Fig. 4 and Table 1.

In addition, based on Eq. (2) and Table 1, it can be said that the higher |T_3_| and lower |T_0_| and |T_2_| resulted in significantly lower Un for OCDs compared to CONs in all frequency bands (except Alpha I). This less energy may be an explanation for a deficiency to move from one state to another (lower neuroplasticity) in the OCD brain.

A detailed analysis of the T_2_ revealed that the link T5-C3 significantly differed in terms of positive and negative occurrence between OCD and CON groups. We think of it as less inhibition imposed by the sensory motor area (C3) on the exaggerated information processed by the cortico- striatal-thalamo-cortical pathway in OCD.

On the other hand, a key role of the balanced triads (T_1_ and T_3_) is the network organization through connecting the modules. The T_1_ and the T_3_ help for integration and segregation in the brain networks by connecting different groups of positively linked nodes with the negative links^39^. Therefore, having higher |T_3_| and consequently more modules (Beta I network) and significantly a smaller number of |T_1_| (Alpha I network) in OCDs could support the idea of less brain functional specialization and disrupted balance between global integration and local specialization in the OCDs.

In this study, the number of dyadic/triadic interactions was reported by (|*P*|, |N|)/(|T_0_|,|T_1_|,|T_2_|,|T_3_|). Based on the results of |*P*|, |N|, |T_i_|s, and TMH metrics, it can be said that although the number of positive and negative links were not statistically different between the brain networks of the OCDs and the CONs, interactions between the links and arrangement of them make a significant difference between the topology of the brain networks of OCDs and healthy subjects. The findings of this study demonstrated that the SBT context and employing higher order interactions (for example, triadic interactions instead of dyadic (individual positive or negative links) ones) as well as topological analysis through hub concept are promising approaches to better understanding the alternations of brain networks in OCDs.

The surprised value *S* results are represented in Supplementary Fig. S1. These results indicate that most of the triadic interactions for most of the subjects follow Heider’s balance theory in all the frequency bands. This means that the number of balanced/unbalanced triads in the brain network is more than those in the random network (more information in the “structural balance” section). However, as presented in Supplementary Table S1, some subjects did not follow Heider’s balance theory mainly for the T_0_ triads. A similar finding has been reported in the previous studies^39^ and some studies of social networks^46,47^ that T_0_ may be over-presented in some cases.

### Limitations and suggestions for future works

Considering the ethical issues, we only recruited OCD patients under medication in our study. However, the medication could influence the functionality of the brain. Therefore, the results may not be directly extended to all OCD patients. Moreover, this study was only based on the resting-state data and further analysis using task-based data is also suggested for future works. A few metrics out of 9 studied metrics were discriminative between OCDs and CONs in different frequency bands. Some of these metrics may be biomarkers for OCD. However, it needs studying much more subjects and can be a topic for the future.

As we said in the discussion, positive links individually and through their interactions with negative and positive links and through making hub offer a stable (low energy) state for OCDs. As OCD characteristics show this stability is along with obsessive thoughts and compulsive behaviors. We think that a treatment approach should attempt to decrease the positive links (increase the negative links) and consequently decrease the positive hubs and T_3_ and increase the T_0_ and T_2_. As a result, energy is injected into the brain which consequently increases the plasticity of OCDs’ brain and helps the brain to change its state. It can be said that such treatment breaks the positive hubs and interactions with positive links and looping thoughts. Looping thoughts are thoughts that are repeated as many as possible in the OCDs’ brain and the treatment can break these loops. Studying such a hypothesis can be an interesting topic for future work and the possible treatments can be noninvasive brain stimulation such as transcranial magnetic stimulation (TMS), transcranial direct current stimulation (tDCS), transcranial alternating current stimulation (tACS), random noise stimulation (RNS), transcranial ultrasound stimulation (TUS)^48^.

The most commonly used EEG devices in clinics include 19 channels (in this study, the data from 18 channels was available). Thus, it was more reachable for us to go for clinical devices and use the data of clinics in research. In addition, these 18 channels were placed based on the 10–20 system, therefore, they fully covered the brain regions. Maybe the number of channels plays a critical role in our findings or the dependency of our findings on channel numbers may be low. The sensitivity of the topology findings of this study to the channel number can be a very interesting topic for future work. For the future, higher channel numbers such as 32, 64, 128, or even 256 can be investigated.

This work attempted to initiate the topological study of OCD versus CON using SBT and TMH concepts and show their promising perspective for such a study. The final verification of our findings needs much more studies with much more subjects and EEG devices with much more channels (investigating the brain with higher spatial resolution and bigger network size).

## Methods

**Figure 3:**
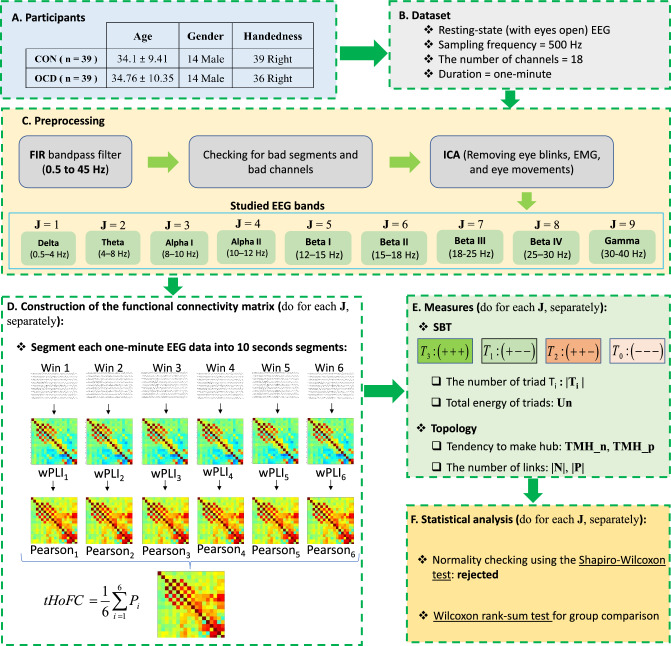
Experimental design. (**A**) Both OCD and CON groups had 39 subjects and matched for age (t(76) = 0.3, *p* = 0.77) and gender (X2(1) = 0, *p* = 1). (**B**) One-minute resting-state EEG dataset was recorded for each subject with a sampling frequency of 500 Hz and 18 electrodes. (**C**) Bandpass filtering and ICA were applied to remove high frequency noise and artifacts. Then, the one-minute data of each subject passed through the nine FIR band-pass filters to have EEG data in nine well-known frequency bands. (**D**) For each subject, the tHOFC matrix was computed for each band, separately. The wPLI and Pearson correlation methods provided the first and second steps of FC matrices for tHOFC, respectively. (**E**) The frequency and total energy of triads were used to compare OCDs and CONs in the perspective of the SBT context. The TMH and frequency of positive and negative links were employed for topological comparison purposes. (**F**) After checking for normal distribution, the Wilcoxon rank-sum test was used for statistical comparison between OCD and CON groups.

A schematic of the experimental design is presented in Fig. 3.

### Participants

The dataset used in this paper was collected in the psychiatric ward of Farshchian Hospital in Hamadan in 2016 (IRCT2015030321313N1, Grant No. 940125303). In this study, 78 subjects were recruited for EEG data collection. Out of the 78 participants, of whom 39 were OCD patients (avg. 34.76 years, std 10.35 years) with the DMS-5 criteria and 39 were healthy individuals (avg. 34.1 years, std 9.41 years). All the study was approved by the ethical committee of the Hamadan University of Medical Sciences. Informed consent was obtained from all subjects. All experiments were performed in accordance with the relevant guidelines and regulations. The following three conditions were considered for the selection of OCD patients; (1) Subjects who had been diagnosed with OCD by a psychiatrist based on the DSM-5 criteria and whose disease had been confirmed based on a clinical interview, (2) Individuals who scored at least 16 on the Yale-Brown obsessive-compulsive scale (Y-BOCS), and (3) People who were in the age range of 18–60 years. In addition, some other participants who met the following conditions were excluded from the study; (1) Any current mental illness except OCD, (2) History of severe head injuries, (3) History of substance dependence (or abuse), (4) Any severe neurological disorder, (5) intellectual impairment, (6) Any electroconvulsive therapy within one year before the study, (7) Any vision, hearing or speech impairment, paralysis, or amputation, and (8) Any clinical condition that has a significant effect on EEG signals (such as pregnancy).

Table 2 summarizes the demographic information of the people along with other parameters. According to this table, *p-values* are greater than the threshold value of 0.05. Therefore, it can be concluded that these parameters (i.e., gender, right-handed or left-handed, and age) do not change significantly among individuals. As a result, the effect of the mentioned parameters is not considered in the analysis.

**Table 2:** Sample characteristics.

	OCD (n = 39)	CON (n = 39)	Statistic	*P*		
Age	34.76 ± 10.35	34.1 ± 9.41	*t(76)=0.3*	*0.77*		
Gender	14 Male	14 Male	*X*^*2*^*(1)=0*	*1*		
Handedness	36 Right	39 Right	*X*^*2*^*(1)=1.38*	*0.24*		

	Medication	n (%)	Dosage range	–	–	–
Medication at time of the study	Sertraline	21 (53.8)	50–150			
	Citalopram	7 (17.9)	20–60			
	Escitalopram	5 (12.9)	10–20			
	Fluoxetine	3 (7.70)	20–80			
	Fluvoxamine	2 (5.1)	50–200			
	Paroxetine	1 (2.6)	20–60			

	Obsession	Compulsion	Total score	–	–	–
Y-BOCS	11.89 ± 2.47	10.41 ± 3.01	22.30 ± 5.11	–	–	–
	Max = 17.00	Max = 18.00	Max = 34.00			
	Min = 8.00	Min = 6.00	Min = 16.00			

The chi-square (X) and t test values and their corresponding probability *P* values are used for group comparison. The variables are reported as mean ± std. The std stands for standard deviation.

Significant values are in italics.

### EEG Dataset

**Figure 4:**
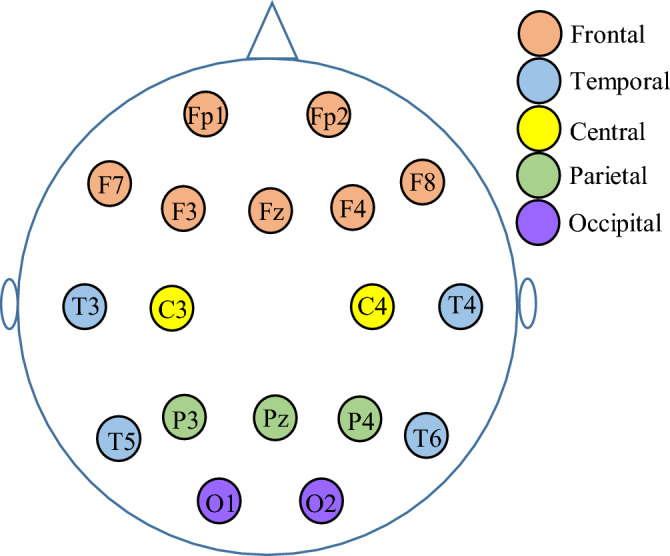
EEG electrode placement based on international 10–20 system. The 18 electrodes cover 5 well-known lobes of the brain, including the Frontal, Temporal, Central, Parietal, and Occipital.

The EEG database was collected from 78 subjects (39 CON cases and 39 OCD patients) using a Cadwell Easy II Amplifier. The scalp electrodes were mounted on the head according to the 10–20 international system. All EEG recordings were collected using 18 Ag–AgCl electrodes. For more details, the positions and names of the electrodes are shown in Fig. 4. All the EEG signals were recorded at a sampling rate of 500 Hz. During the recording, the subjects (with eyes open) were in a resting state. The electrode impedance was less than 5 kΩ during the experiment.

### Preprocessing

At first, the raw one-minute EEG signals were bandpass filtered using a basic finite impulse response (FIR) filter with low and high cut-off frequencies of 0.5 Hz and 45 Hz, respectively. All EEG signal recordings were investigated for the bad segments and bad channels. Fortunately, all data had a satisfactory quality. Then, the artifacts, i.e., eye blinks, horizontal and vertical eye movements and electromyogram (EMG) were eliminated using the independent component analysis (ICA) technique and visual inspection. Afterward, the FIR bandpass filter was applied to filter the one-minute EEG signals in nine sub-bands, including Delta (0.5–4 Hz), Theta (4–8 Hz), Alpha I (8–10 Hz), Alpha II (10–12 Hz), Beta I (12–15 Hz), Beta II (15–18 Hz), Beta III (18–25 Hz), Beta IV (25–30 Hz), and Gama (30–40 Hz). In each of these bands, the interested metrics were computed and compared between OCD and CON.

### Functional connectivity matrix

Among the neuroimaging techniques, functional connectivity (FC) is one of the most popular frameworks in which researchers examine patterns of communication between time-series recorded by electrodes. FC matrix is needed to analyze the triadic interactions and topological impact of positive and negative links in the brain. For each subject and in each frequency band, the one-minute signal was divided into six separate segments of nine seconds. Then, the FC matrix was computed for each segment, separately. The final FC matrix was obtained by averaging the six FC matrices. In the following, it is explained how to compute the FC matrix of each segment.

In this study, the topographical profile similarity-based high-order FC (tHOFC), which is a two-step procedure, was used to compute the FC matrix^49^. In the first step, the FC was computed between each pair of electrodes to be formed the low-order FC (LOFC) matrix. By doing so, a vector of FCs was obtained for each electrode. This vector represented the LOFC profile of the given electrode. In the second step, tHOFC was computed as the similarity of LOFC profiles between each pair of electrodes. The tHOFC can offer complementary information to the conventional LOFC and introduce more differences between groups^49,50^.

For the first step, the weighted phase lag index (wPLI) was used^51^. The wPLI is very popular in EEG modality due to its better test–retest reliability as compared to other functional connectivity measures^52^. The wPLI like PLI measures the phase angle differences between the time series of two electrodes x and y using the imaginary part of cross-spectral density *S*_*xy*_ of two electrodes’ signals. The wPLI in comparison to PLI employs a more conservative approach to deal with potential confounds caused by volume conduction, by scaling contributions of angle differences according to their distance from the real axis:

1 $$ wPLI = \left| {\frac{{\sum\limits_{t = 1}^{n} {\left| {imag(S_{xy,t} )} \right|{\text{sgn}} (imag(S_{xy,t} ))} }}{{\sum\limits_{t = 1}^{n} {\left| {imag(S_{xy,t} )} \right|} }}} \right| $$

where |.| indicates the absolute value, sgn is the sign function, *imag* represents the imaginary part, and *t* and *n* are the time index and the total number of time points, respectively.

For triadic interaction analysis, FC matrices must have both positive and negative values. Hence, for the second step, the Pearson correlation was used to provide a similarity profile and final FC matrix for each segment.

### The number of links

The number (or occurrence rate) of negative links |N| and positive links |*P*| are computed for each subject in each frequency band. By having the information of |N| and |*P*| it can be answered that |T_i_|s and TMHs may become different between groups even when there is no difference in terms of the number of negative and positive links.

### Structural balance

**Figure 5:**
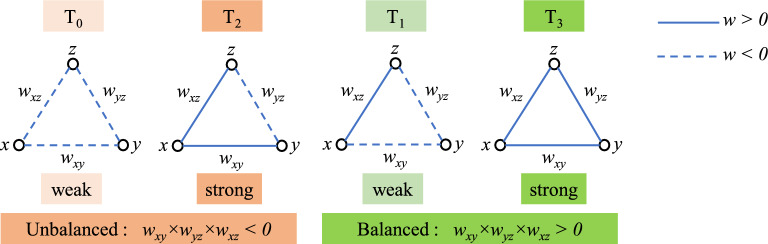
Four types of triads are defined by SBT. The number of negative links is even/odd for balanced/unbalanced triads. The subscript of each T denotes the number of positive links. For balanced triads, the strong vs weak means that T_3_ is more stable than T_1_. For unbalanced triads, the strong triad T_2_ injects more frustration into the network than the weak triad T_0_.

SBT investigates the stability and behavior of a network by analyzing the triadic interactions between entities (herein electrodes). In this study, as with Moradimanesh and colleagues^39^, four types of triads were analyzed in nine frequency bands. These triads were strongly balanced T_3_: (+ + +), weakly balanced T_1_: (+ − −), strongly unbalanced T_2_: (+ + −), and weakly unbalanced T_0_: (− − −) (Fig. 5). The “+” and “−“ are the sign of FC weights between the electrodes and, respectively, represent the phase synchrony and asynchrony between electrodes (brain regions). This way, information about the organization of the network that cannot be detected on the level of pair connections would get a chance to be revealed. A well-known analogy for this definition is that positive (negative) links are considered friendship (enmity) relations, respectively. Then, the classic balance model defines a triad as balanced if it contains no violations of four assumptions: (A1) A friend of a friend is a friend, (A2) A friend of an enemy is an enemy, (A3) An enemy of a friend is an enemy, (A4) An enemy of an enemy is a friend^40,53,54^. The first assumption is represented by T_3_ and the rest assumptions are represented by T_1_. Otherwise, we have unbalanced triads, T_2_ (“a friend’s friend is an enemy”) and T_0_ (“an enemy’s enemy is an enemy”)^40,53,54^. Entities of an unbalanced triad are frustrated about their conditions and try to change their relationships to reach one of the balanced conditions. This is analogous to the transition of an unstable physical system toward a stable state with a lower energy level based on the principle of minimum energy. Accordingly, it can be assumed that a balanced (stable) triad situates stationary in a low-level energy state despite an imbalanced (unstable) triad that tries to solve its frustrations to reduce its energy and move to the stable state^40,55^. The T_2_ and T_0_ respectively are strong and weak unbalanced triads (or equally strong and weak frustrated triads) because the T_2_ injects more frustration into the system than T_0_ (the entities of T_2_ are more frustrated than those of T_0_ about their conditions and, therefore, the T_2_ is more unbalance (unstable) than T_0_). Likewise, the T_3_ and T_1_ are strong and weak balanced triads because a T_3_ is more stable than T_1_ and a system containing entirely triple positive triads is more stable^35,40^.

The balanced (stable) state of a network is a state in which there is no frustration and all triads are balanced (stable) ones. Based on the principle of minimum energy, a stable network (or a network in a balanced state) has the least energy and remains stationary since there is no dynamic demand to change link signs due to the presence of imbalanced triads^40,56^. Therefore, one should know the definition of balance-energy to find the balanced state of a network. For a balanced interaction, the product of its edges is a positive number, whereas, for an unbalanced interaction, this product is negative. As previously proposed^57^, if one sums the negative of these products and divides it by the total number of ternary interactions, the total energy Un of a network would be obtained. Energy represents the extent to which a network is structurally balanced. This definition of energy is similar to the Hamiltonian concept in physics. The Hamiltonian of a system represents the total energy of the system, that is, the sum of the energies of all particles (triads) associated with the system^58^. The negative of products for computation of Un helps to better understand the equation from the physical energy perspective. This means that the balanced/unbalanced triads make the network stable/unstable. Thus, products of their components should decrease/increase the energy of the network. To achieve this property, the negative of products was proposed by Marvel and colleagues^57^. The total energy of network Un is defined as

2 $$ Un = - \frac{1}{N}\sum\limits_{i = 0}^{3} {\sum\limits_{x < y < z} {w_{xy} (T_{i} )w_{xz} (T_{i} )w_{yz} (T_{i} )} } $$

where *N* is the total number of network triads, *w* is the edge weight of triad T_i_, and *x*, *y*, and *z* indicate to electrodes of triad T_i_. As with^39,40^, we call this total energy balance-energy because based on which one can understand how much a network is balanced and how (i.e., positively or negatively) the metrics impact the network stability by analyzing the relation between Un and those metrics. For a fully strong (strong means weights are + 1 and − 1) balanced and unbalanced network, the Un would be − 1 and + 1, respectively. Therefore, the minimization of Un leads to more stability of the network.

If one variable or metric decreases the Un, (based on the definition given in this subsection) one can say that that metric leads to a more balanced state for the network. For example, in this study, the results show that the tendency of OCDs’ brains for making hubs with positive links (the tendency to make hubs is a metric explained in the next subsection) is significantly more than that of CONs’ brains in many frequency bands. Also, this tendency has a much stronger anti-correlation with Un for OCDs in comparison to CONs in all frequency bands. As a result, one can conclude that such a tendency leads to a more balanced (lower energy Un) state for the OCDs’ brains.

In this study, two metrics were used to compare the triadic interactions between OCD and CON. The first one was the occurrence rate of triad T_i_ (indicating by |T_i_|) where i = 1, 2, 3, 4. In this paper, the occurrence rate of something such as T_i_ or a link of T_i_ is equal to the number of times it happens. The second one was the Un.

It is expected that the balanced/unbalanced triads become over-/under-presented in the brain network. This means that the number of balanced/unbalanced triads in the brain network is more than those in the random network. The surprised value *S* is a metric providing the positive/negative values for over-/under-presented triads^39^:

3 $$ S(T_{i} ) = (|T_{i} | - p_{0} (T_{i} ))/\sqrt {Np_{0} (T_{i} )(1 - p_{0} (T_{i} ))} $$

where the $p_{0} (T_{i} )$ is the ratio of the |T_i_| to the total number of triads in the random network. The random network has the same links as the brain network and only the signs of brain network links are randomly assigned to the existing links of the random network. In this study, the* S* was only used to provide information for the over-/under-presented behavior of triads in OCD and CON, and not for group comparison.

### Tendency to make hub

Hubs are nodes (electrodes) with a high number of connections and these connections have high weights in weighted networks. Hubs, which are local features of networks, play a key role in the topology of the brain network. In this study, we used the global hubness metric to compare the brain networks of OCDs with CONs in an aspect of network topology. This metric was first time introduced by Saberi and colleagues and used to study autism spectrum disorder^40^. This metric, which is named the tendency to make hub (TMH), is separately defined for positive and negative links as TMH_p and TMH_n:

4 $$ TMH\_p = \frac{{\sum\limits_{i = 1}^{M} {D_{i,p}^{2} } }}{{\sum\limits_{i = 1}^{M} {D_{i,p} } }},D_{i,p} = \sum\limits_{{j = 1,j \ne i,,w_{ij} > 0}}^{M} {w_{ij,p} } ;TMH\_n = \frac{{\sum\limits_{i = 1}^{M} {D_{i,n}^{2} } }}{{\sum\limits_{i = 1}^{M} {D_{i,n} } }},D_{i,n} = - \sum\limits_{{j = 1,j \ne i,w_{ij} < 0}}^{M} {w_{ij,n} } $$

where *M* is the total number of electrodes, *D*_*i,p*_ and *D*_*i,n*_ represent the positive and negative degrees of ith electrode, respectively, and *w*_*ij,p*_ and *w*_*ij,n*_ are the positive and negative weights between ith electrode and the rest of the electrodes.

The TMH_p and TMH_n demonstrate the tendency of the network to make hubs with positive and negative links, separately. Thus, the TMH can describe the impact of positive and negative links on the topology of the brain.

### Statistical analysis

First of all, the number of positive and negative links and their ratio were compared between OCD and CON groups. Subsequently, the number of balanced and unbalanced triads were compared and the structural balance-energy of the networks in OCD and CON groups were examined. Then, considering no significant differences in terms of the total number of positive and negative links between the groups, the effect of the topology of the links was investigated by comparing between global tendency in the network to make positive and negative hubs. The above-mentioned comparisons between metrics of OCD and CON were performed for the networks of Delta, Theta, Alpha I, Alpha II, Beta I, Beta II, Beta III, Beta IV, and Gamma bands separately. In each of these bands, the group-level normality of the measures was tested using the Shapiro–Wilk test^59^. The results showed that the distributions of some measures at some frequency bands were far from normal. Therefore, the Wilcoxon rank-sum test as a nonparametric test was used for statistical comparison purposes^60^. A threshold of *p* < *0.05* (Fisher permutation) was considered to find the statistically significant differences between OCD and CON groups. We used the Bonferroni method^61^ to correct the results for multiple comparison effects. A threshold of *P* value < (0.05/(18*18*9)) was considered for the number of positive and negative links. Since the other metrics are global, the correction was only performed for the frequency bands and a threshold of *P* value < (0.05/9) was implied.

## Supplementary Information

Supplementary Information.

## Data Availability

The datasets and codes used during the current study are available from the corresponding author upon request.

## References

[1] American Psychiatric Association (2013). Diagnostic and Statistical Manual of Mental Disorders.

[2] Abramovitch A, Abramowitz JS, Mittelman A (2013). The neuropsychology of adult obsessive-compulsive disorder: A meta-analysis. Clin. Psychol. Rev..

[3] Yazdi-Ravandi S (2018). Differential pattern of brain functional connectome in obsessive-compulsive disorder versus healthy controls. EXCLI J..

[4] Greisberg S, McKay D (2003). Neuropsychology of obsessive-compulsive disorder: A review and treatment implications. Clin. Psychol. Rev..

[5] Keefe RS (1995). The contribution of neuropsychology to psychiatry. Am. J. Psych..

[6] Kuelz AK, Hohagen F, Voderholzer U (2004). Neuropsychological performance in obsessive-compulsive disorder: A critical revie. Biol. Psychol..

[7] Rao NP, Reddy YC, Kumar KJ, Kandavel T, Chandrashekar CR (2008). Are neuropsychological deficits trait markers in OCD?. Prog. Neuropsychopharmacol. Biol. Psych..

[8] Shin MS, Choi H, Kim H, Hwang JW, Kim BN, Cho SC (2008). A study of neuropsychological deficit in children with obsessive-compulsive disorder. Eur. Psych..

[9] Ruscio AM, Stein DJ, Chiu WT, Kessler RC (2010). The epidemiology of obsessive-compulsive disorder in the National Comorbidity Survey Replication. Mol. Psych..

[10] Benzina N, Mallet L, Burguière E, N’diaye K, Pelissolo A (2016). Cognitive dysfunction in obsessive-compulsive disorder. Curr. Psych. Rep..

[11] Friedlander L, Desrocher M (2006). Neuroimaging studies of obsessive-compulsive disorder in adults and children. Clin. Psychol. Rev..

[12] Lewin AB, Larson MJ, Park JM, McGuire JF, Murphy TK, Storch EA (2014). Neuropsychological functioning in youth with obsessive compulsive disorder: An examination of executive function and memory impairment. Psych. Res..

[13] Radmanesh A, Zaman T, Ghanaati H, Molaei S, Robertson RL, Zamani AA (2008). Methylmalonic acidemia: Brain imaging findings in 52 children and a review of the literature. Pediatr. Radiol..

[14] Zhuo C, Zhu J, Wang C, Qu H, Ma X, Qin W (2017). Different spatial patterns of brain atrophy and global functional connectivity impairments in major depressive disorder. Brain Imaging Behav..

[15] Ferrari MCF, Busatto GF, McGuire PK, Crippa JAS (2008). Structural magnetic ressonance imaging in anxiety disorders: An update of research findings. Braz. J. Psych..

[16] Parmar A, Sarkar S (2016). Neuroimaging studies in obsessive compulsive disorder: A narrative review. Indian J. Psychol. Med..

[17] Robinson D, Wu H, Munne RA, Ashtari M, Alvir JMJ, Lerner G, Bogerts B (1995). Reduced caudate nucleus volume in obsessive-compulsive disorder. Arch. Gen. Psych..

[18] Szeszko PR (1999). Orbital frontal and amygdala volume reductions in obsessive-compulsive disorder. Arch. Gen. Psych..

[19] Bédard MJ, Joyal CC, Godbout L, Chantal S (2009). Executive functions and the obsessive-compulsive disorder: On the importance of subclinical symptoms and other concomitant factors. Arch. Clin. Neuropsychol..

[20] Burguiere E, Monteiro P, Mallet L, Feng G, Graybiel AM (2015). Striatal circuits, habits, and implications for obsessive-compulsive disorder. Curr. Opin. Neurobiol..

[21] Ham T, Leff A, de Boissezon X, Joffe A, Sharp DJ (2013). Cognitive control and the salience network: An investigation of error processing and effective connectivity. J. Neurosci..

[22] Ortigue S, Patel N, Bianchi-Demicheli F (2009). New electroencephalogram (EEG) neuroimaging methods of analyzing brain activity applicable to the study of human sexual response. J. Sex. Med..

[23] Bucci P, Mucci A, Volpe U, Merlotti E, Galderisi S, Maj M (2004). Executive hypercontrol in obsessive-compulsive disorder: Electrophysiological and neuropsychological indices. Clin. Neurophysiol..

[24] Locatelli M, Bellodi L, Grassi B, Scarone S (1996). EEG power modifications in obsessive-compulsive disorder during olfactory stimulation. Biol. Psych..

[25] Prichep LS (1993). Quantitative electroencephalographic subtyping of obsessive-compulsive disorder. Psych. Res. Neuroimaging..

[26] Yazdi-Ravandi S (2021). Complexity of information processing in obsessive-compulsive disorder based on fractal analysis of EEG signal. EXCLI J..

[27] Ischebeck M, Endrass T, Simon D, Kathmann N (2014). Altered frontal EEG asymmetry in obsessive-compulsive disorder. Psychophysiology.

[28] Morand-Beaulieu S, Aardema F, O'Connor KP, Lavoie ME (2021). Lateralized readiness potentials and sensorimotor activity in adults with obsessive-compulsive disorder. Prog. Neuropsychopharmacol. Biol. Psych..

[29] Wong M, Woody EZ, Schmidt LA, Ameringen MV, Soreni N, Szechtman H (2015). Frontal EEG alpha activity and obsessive-compulsive behaviors in non-clinical young adults: A pilot study. Front. Psychol..

[30] Watts DJ, Strogatz SH (1998). Collective dynamics of ‘small-world’networks. Nature.

[31] Bullmore E, Sporns O (2009). Complex brain networks: Graph theoretical analysis of structural and functional systems. Nat. Rev. Neurosci..

[32] Sporns O (2010). Networks of the Brain.

[33] Long Z, Duan X, Mantini D, Chen H (2016). Alteration of functional connectivity in autism spectrum disorder: Effect of age and anatomical distance. Sci. Rep..

[34] Sadeghi M, Khosrowabadi R, Bakouie F, Mahdavi H, Eslahchi C, Pouretemad H (2017). Screening of autism based on task-free fmri using graph theoretical approach. Psych. Res. Neuroimaging..

[35] Belaza AM, Hoefman K, Ryckebusch J, Bramson A, Van Den Heuvel M, Schoors K (2017). Statistical physics of balance theory. PLoS ONE.

[36] Doreian P, Mrvar A (2019). Structural balance and signed international relations. J. Soc. Struct..

[37] Saiz H, Gómez-Gardeñes J, Nuche P, Girón A, Pueyo Y, Alados CL (2017). Evidence of structural balance in spatial ecological networks. Ecography.

[38] Cartwright D, Harary F (1956). Structural balance: A generalization of Heider’s theory. Psychol. Rev..

[39] Moradimanesh Z, Khosrowabadi R, Gordji ME, Jafari GR (2021). Altered structural balance of resting-state networks in autism. Sci. Rep..

[40] Saberi M, Khosrowabadi R, Khatibi A, Misic B, Jafari G (2021). Topological impact of negative links on the stability of resting-state brain network. Sci. Rep..

[41] Geffen T, Smallwood J, Finke C, Olbrich S, Sjoerds Z, Schlagenhauf F (2022). Functional connectivity alterations between default mode network and occipital cortex in patients with obsessive-compulsive disorder (OCD). NeuroImage Clin..

[42] Stern ER, Fitzgerald KD, Welsh RC, Abelson JL, Taylor SF (2012). Resting-state functional connectivity between fronto-parietal and default mode networks in obsessive-compulsive disorder. PLoS ONE.

[43] Luo L, Li Q, You W, Wang Y, Tang W, Li B, Gong Q (2021). Altered brain functional network dynamics in obsessive-compulsive disorder. Hum. Brain Mapp..

[44] Saberi M, Khosrowabadi R, Khatibi A, Misic B, Jafari G (2021). Requirement to change of functional brain network across the lifespan. PLoS ONE.

[45] Heider F (1982). The Psychology of Interpersonal Relations.

[46] Szell M, Lambiotte R, Thurner S (2010). Multirelational organization of large-scale social networks in an online world. Proc. Natl. Acad. Sci..

[47] Leskovec, J., Huttenlocher, D., & Kleinberg, J. Signed networks in social media. In *Proceedings of the SIGCHI Conference on Human Factors in Computing Systems*, pp. 1361–1370 (2010).

[48] Bhattacharya A, Mrudula K, Sreepada SS, Sathyaprabha TN, Pal PK, Chen R, Udupa K (2022). An overview of noninvasive brain stimulation: Basic principles and clinical applications. Can. J. Neurol. Sci..

[49] Zhou Z (2020). A toolbox for brain network construction and classification (BrainNetClass). Hum. Brain Mapp..

[50] Zhang H, Giannakopoulos P, Haller S, Lee S-W, Qiu S, Shen D (2019). Inter-network high-order functional connectivity (in-hofc) and its alteration in patients with mild cognitive impairment. Neuroinformatics.

[51] Vinck M, Oostenveld R, Van Wingerden M, Battaglia F, Pennartz CM (2011). An improved index of phase-synchronization for electrophysiological data in the presence of volume-conduction, noise and sample-size bias. Neuroimage.

[52] Hardmeier M, Hatz F, Bousleiman H, Schindler C, Stam CJ, Fuhr P (2014). Reproducibility of functional connectivity and graph measures based on the phase lag index (PLI) and weighted phase lag index (wPLI) derived from high resolution EEG. PLoS ONE.

[53] Rawlings CM, Friedkin NE (2017). The structural balance theory of sentiment networks: Elaboration and test. Am. J. Sociol..

[54] Rapoport, A. Mathematical models of social interaction. In *Handbook of Mathematical Psychology*, Vol. II. (Wiley, 1963).

[55] Antal T, Krapivsky PL, Redner S (2005). Dynamics of social balance on networks. Phys. Rev. E..

[56] Callen HB (1960). Thermodynamics and an Introduction to Thermostatistics.

[57] Marvel SA, Strogatz SH, Kleinberg JM (2009). Energy landscape of social balance. Phys. Rev. Lett..

[58] Van der Schaft AJ (1986). Stabilization of Hamiltonian systems. Nonlinear Anal. Theory Methods Applic..

[59] Shapiro SS, Wilk MB (1965). An analysis of variance test for normality (complete samples). Biometrika.

[60] Mann HB, Whitney DR (1947). On a test of whether one of two random variables is stochastically larger than the other. Ann. Math. Stat..

[61] Ludbrook J (1998). Multiple comparison procedures updated. Clin. Exp. Pharmacol. Physiol..

