# Supplementary material for: Balance-energy of resting state network in obsessive-compulsive disorder
Source: Sci Rep. 2023 Jun 27;13:10423. doi: 10.1038/s41598-023-37304-9 (PMC10300010; doi:10.1038/s41598-023-37304-9)
Supplement: Supplementary file 1 — Supplementary Information. [file 41598_2023_37304_MOESM1_ESM.pdf]

Supplementary Information

Balance Energy of Resting State Network in Obsessive-Compulsive Disorder

Alireza Talesh[1], Asghar Zarei[1], Saeid Yazdi-Ravandi[2*], Ali Ghaleiha[2], Farshid Shamsaei[2], Nasrin Matinnia[3], Jamal Shams[4], Mohammad Ahmadpanah[2], Zahra Taslimi[5], Abbas Moghimbeigi[6], Reza Khosrowabadi[7]*

Affiliations:

[1] Department of Biomedical Engineering, Tarbiat Modares University, Tehran, Iran.

[2] Behavioral Disorders and Substance Abuse Research Center, Hamadan University of Medical Sciences, Hamadan, Iran

[3] Department of Nursing, College of Basic Science, Hamadan Branch, Islamic Azad University, Hamadan, Iran

[4] Behavioral ScienBces Research Center, Shahid Beheshti University of Medical Sciences, Tehran, Iran

[5] Neurophysiology Research Center, Hamadan University of Medical Sciences, Hamadan, Iran

[6] Modeling of Noncommunicable Disease Research Center, Department of Biostatistics, School of Public Health, Hamadan University of Medical Sciences, Hamadan, Iran

[7] Institute for Cognitive and Brain Sciences, Shahid Beheshti University, Tehran, Iran.

S.1. Results of surprise value *S*

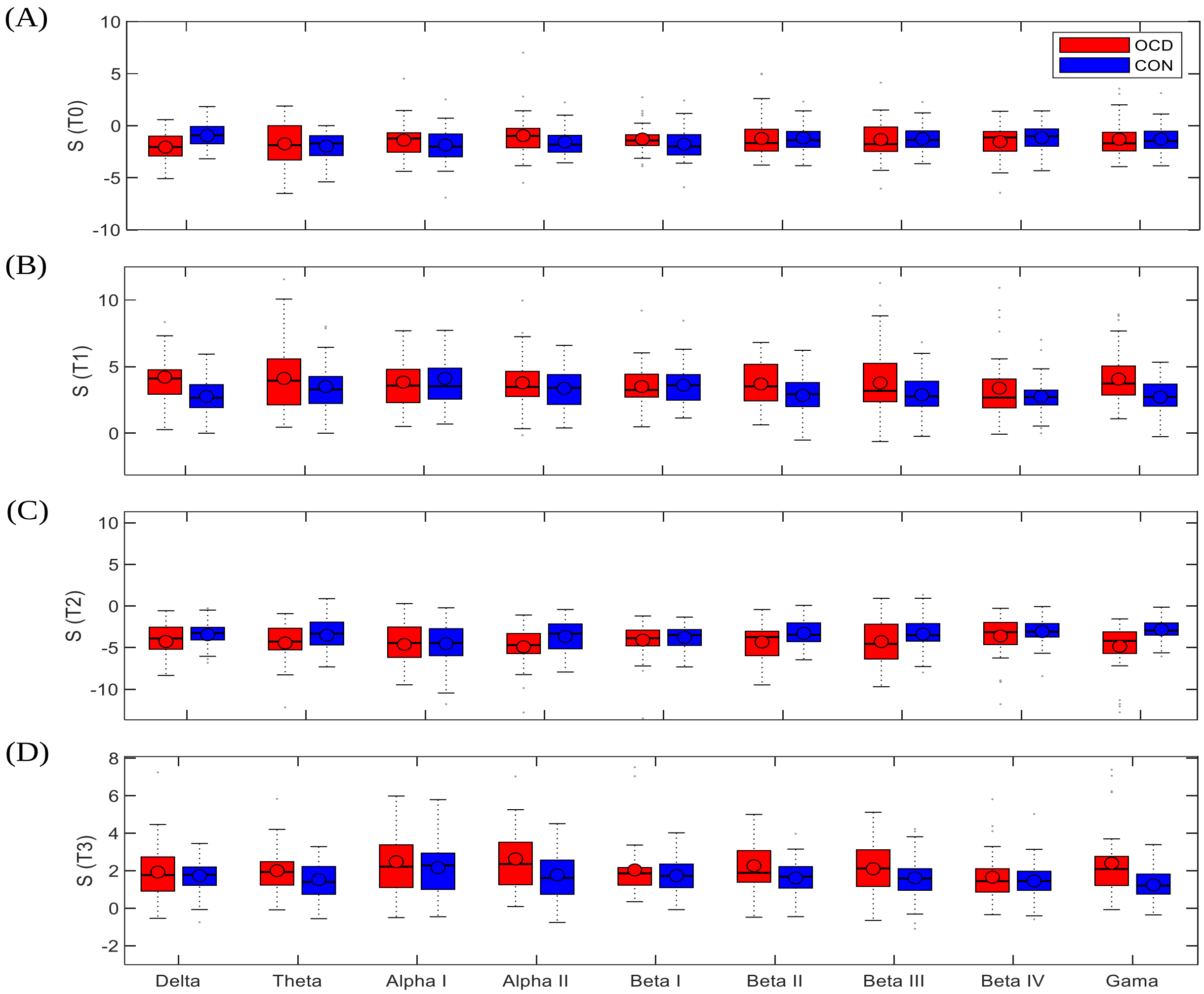

Supplementary Figure S1. The boxplots of surprised values *S* of T_0, T_1, T_2, and T_3 in nine well-known frequency bands of EEG. Overall, for both groups, the values of *S* are positive/negative for balanced/unbalanced triads.

S.2. The number of subjects that did not follow Heider’s balance theory

Supplementary Table S1. The number of subjects that did not follow Heider’s balance theory. Based on Heider’s balance theory, the unbalanced/balanced triads are over-/under-presented in the network. The unbalanced triad T_0 has the maximum number of subjects for both OCD and CON groups.

		Delta	Theta	Alpha I	Alpha II	Beta I	Beta II	Beta III	Beta IV	Gama
**T_0**	OCD	3	8	4	7	5	5	9	5	7
	CON	9	0	5	6	5	9	7	6	6
**T_1**	OCD	0	0	0	1	0	0	1	1	0
	CON	0	0	0	0	0	2	1	0	2
**T_2**	OCD	0	0	1	0	0	0	3	0	0
	CON	0	1	0	0	0	1	3	0	0
**T_3**	OCD	2	2	1	0	0	1	4	2	1
	CON	2	2	2	1	1	2	4	3	3
